# The role of O-GlcNAcylation in innate immunity and inflammation

**DOI:** 10.1093/jmcb/mjac065

**Published:** 2022-12-06

**Authors:** Yongqiang Wang, Xiuwu Fang, Shuai Wang, Bin Wang, Feng Chu, Zhixin Tian, Long Zhang, Fangfang Zhou

**Affiliations:** Institutes of Biology and Medical Science, Soochow University, Suzhou 215123, China; Institutes of Biology and Medical Science, Soochow University, Suzhou 215123, China; Institutes of Biology and Medical Science, Soochow University, Suzhou 215123, China; MOE Laboratory of Biosystems Homeostasis and Protection and Innovation Center for Cell Signaling Network, Life Sciences Institute, Zhejiang University, Hangzhou 310058, China; Institutes of Biology and Medical Science, Soochow University, Suzhou 215123, China; Institutes of Biology and Medical Science, Soochow University, Suzhou 215123, China; MOE Laboratory of Biosystems Homeostasis and Protection and Innovation Center for Cell Signaling Network, Life Sciences Institute, Zhejiang University, Hangzhou 310058, China; Institutes of Biology and Medical Science, Soochow University, Suzhou 215123, China

**Keywords:** innate immunity, inflammation, O-GlcNAcylation, cancer

## Abstract

O-linked β-*N*-acetylglucosaminylation (O-GlcNAcylation) is a highly dynamic and widespread post-translational modification (PTM) that regulates the activity, subcellular localization, and stability of target proteins. O-GlcNAcylation is a reversible PTM controlled by two cycling enzymes: O-linked *N*-acetylglucosamine transferase and O-GlcNAcase. Emerging evidence indicates that O-GlcNAcylation plays critical roles in innate immunity, inflammatory signaling, and cancer development. O-GlcNAcylation usually occurs on serine/threonine residues, where it interacts with other PTMs, such as phosphorylation. Thus, it likely has a broad regulatory scope. This review discusses the recent research advances regarding the regulatory roles of O-GlcNAcylation in innate immunity and inflammation. A more comprehensive understanding of O-GlcNAcylation could help to optimize therapeutic strategies regarding inflammatory diseases and cancer.

## Introduction

In 1984, Torres and Hart (1984) first reported the modification of cell surface proteins in lymphocytes by O-linked β-*N*-acetylglucosamine (O-GlcNAc). Following this discovery, O-GlcNAc has been reported to modify a wide variety of proteins (Torres and Hart, 1984). Subsequently, O-GlcNAcylation (the process by which O-GlcNAc moieties are conjugated to proteins) has been shown to occur in some bacteria, protozoa, fungi, and viruses, as well as in all metazoans; however, it has not yet been observed in yeast (Hu et al., 2010). O-GlcNAcylation is an essential cellular regulator of many biochemical processes, including stress protection, nutrient sensing, cell cycle progression, protein–protein interactions, protein transcription, translation, and degradation, and signaling pathways (Hart, 1997; Comer and Hart, 2000; Hart et al., 2007). Classical protein glycosylation is considered to constitute a stable and form of conserved modification (Schjoldager et al., 2020). However, O-GlcNAc exists exclusively on nuclear and cytoplasmic proteins. It does not become modified or elongated to form more complex structures, unlike the complex arrays of glycan on extracellular glycoproteins. Moreover, O-GlcNAcylation is highly dynamic; it features rapid cycling in response to different physiological stimuli (analogous to phosphorylation) (Hart et al., 2007). Although the consensus motif for O-GlcNAcylation has not yet been identified, serine and threonine kinases are known to frequently use many O-GlcNAcylation sites. This suggests that O-GlcNAcylation may function together with phosphorylation to regulate protein activity and signal transduction (Cheng and Hart, 2001; Comer and Hart, 2001; Walgren et al., 2003). In addition, many studies have revealed the deregulation of O-GlcNAcylation to be associated with various diseases in humans, such as diabetes, neurodegenerative diseases, cardiovascular diseases, and cancer (Copeland et al., 2008; Slawson and Hart, 2011; Bond and Hanover, 2013; Wright et al., 2017). Therefore, a better understanding of O-GlcNAcylation could help to reveal potential drug targets for the treatment of these diseases.

This review describes the basic principles of O-GlcNAcylation and highlights its important roles in viral infections and inflammatory signaling. Moreover, the regulation of intracellular O-GlcNAc is discussed herein, as are the implications of its dysfunction in cancer, and the phenomenon of crosstalk between O-GlcNAcylation and other post-translational modifications (PTMs). A more comprehensive understanding of O-GlcNAcylation is crucial to the development of effective cancer therapeutics.

## Process of O-GlcNAcylation

**Figure 1:**
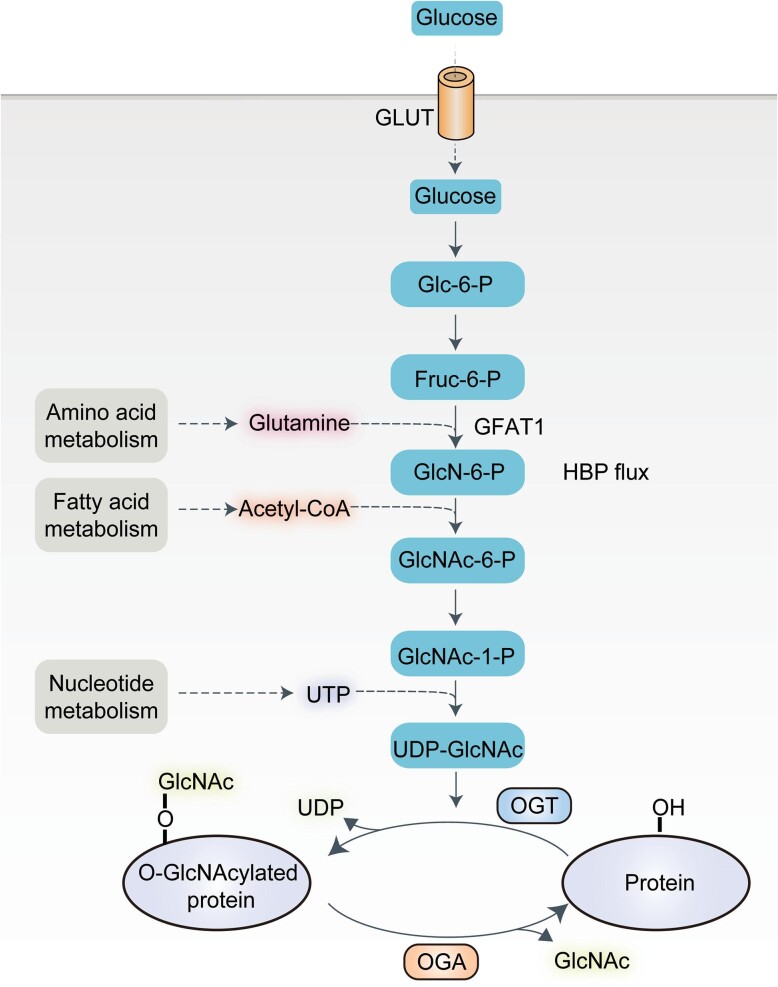
The hexosamine biosynthetic pathway regulates protein O-GlcNAcylation. After glucose is taken up into cells through the transporter GLUT protein, ∼2%–5% of it enters the HBP pathway for metabolism. Fructose-6-phosphate (Fruc-6-P) amidotransferase 1 (GFAT1) is the rate-limiting enzyme of HBP; it catalyzes the conversion of Fruc-6-P to glucosamine-6-phosphate (GlcN-6-P). GlcN-6-P then undergoes acetylation and uridylation to generate the O-GlcNAcylation substrate UDP-GlcNAc. OGT and OGA add and remove O-GlcNAc from protein, respectively.

Protein O-GlcNAcylation is catalyzed by a single pair of enzymes, O-linked *N*-acetylglucosamine transferase (OGT) and O-GlcNAcase (OGA); OGT catalyzes the addition of O-GlcNAc onto proteins, while OGA has the opposite effect (Yang and Qian, 2017). The donor substrate of OGT is uridine diphosphate *N*-acetylglucosamine (UDP-GlcNAc), which is the final product of the hexosamine biosynthetic pathway (HBP) (Figure 1; Chatham et al., 2008). The HBP is one of several glucose metabolism pathways that occur in various tissues throughout the body. In its resting state, ∼2%–5% of glucose is shunted via the HBP; most of this glucose is metabolized through glycolysis or via the synthesis of glycogen and fat (Nie and Yi, 2019). Several metabolic cellular pathways are associated with the HBP, such as the glucose, amino acid, fatty acid, and nucleotide metabolic pathways; these pathways converge to form UDP-GlcNAc (Chatham et al., 2008; Ma and Vosseller, 2014; Myslicki et al., 2014).

OGT is a soluble enzyme that is found in the cytoplasm and nuclei of metazoan cells (Janetzko and Walker, 2014). In mammals, OGT produces three isoforms through alternative splicing: nucleocytoplasmic OGT (ncOGT), mitochondrial OGT (mOGT), and short OGT (sOGT) (Li et al., 2019b). These isoforms share a common catalytic and phosphorylase-derived C-terminal domain, but they vary in length due to their differing numbers of N-terminal tetratricopeptide repeats. Interestingly, these three OGT isoforms are known to vary in their localization; ncOGT and sOGT are located in the cytoplasm and nucleus, whereas mOGT is found in the mitochondria (Ruan et al., 2013; Bond and Hanover, 2015). The most well-established role of OGT entails adding β-O-GlcNAc to the serine and threonine residues of nuclear and cytoplasmic proteins. However, it also exerts other biochemical functions, such as catalyzing site-specific proteolysis. Furthermore, it serves as an integral component of several protein complexes (Levine and Walker, 2016).

OGA hydrolyzes O-GlcNAc from a broad range of O-GlcNAcylated proteins and then returns them to their unmodified state (Li et al., 2017a). From an evolutionary standpoint, conserved OGA exists in two isoforms: longer OGA (OGA-L), which is present in the cytoplasm and nucleus, and shorter OGA (OGA-S). OGA-L contains an N-terminal, which is a member of the GH84 family of glycoside hydrolases, and a C-terminal, comprising the histone acetyltransferase-like (HAT-like) domain (Vocadlo, 2012). However, OGA-S lacks this C-terminal domain; moreover, it is predominantly located in the endoplasmic reticulum and in lipid droplets (Comtesse et al., 2001). The addition and removal of O-GlcNAc by OGT and OGA, respectively, contribute to the dynamic and continuous cycling of O-GlcNAc (Hanover et al., 2012).

## Role of O-GlcNAcylation in the RLR signaling pathway

**Figure 2:**
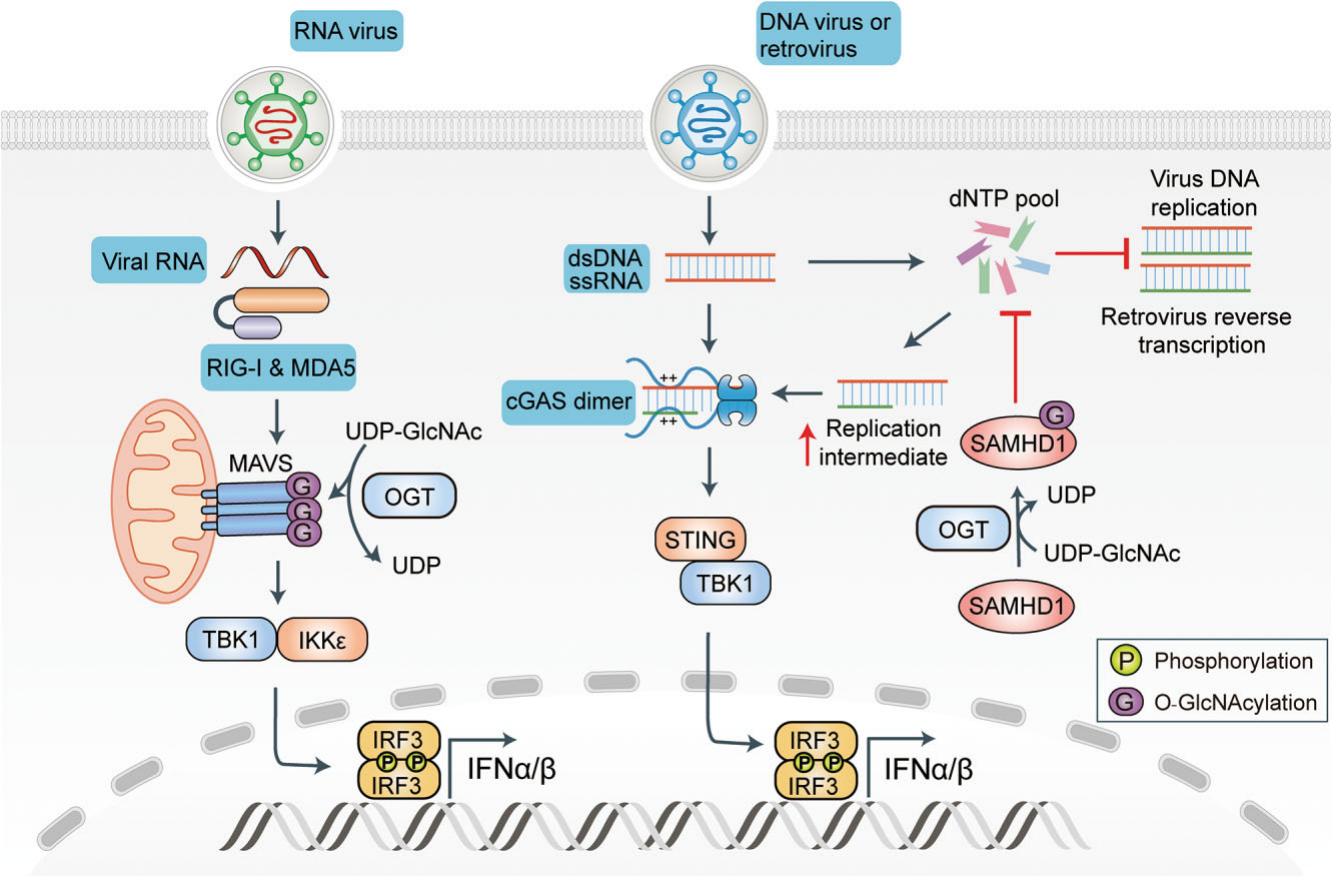
Protein O-GlcNAcylation in the RLR and DNA sensor-mediated signaling pathways. RNA viruses are recognized by RIG-I and MDA5; they then recruit and activate downstream MAVS. Activated MAVS recruits and activates downstream TBK1 and IKKε, resulting in activation of IRF3 and subsequent production of type I interferons (IFNs). O-GlcNAcylation plays an important role in regulating MAVS activation and antiviral signal transduction. After infection by a DNA virus or retrovirus, the virus proliferates through DNA replication or RNA reverse transcription. O-GlcNAcylation of SAMHD1 promotes depletion of the intracellular dNTP pool, resulting in the production of more viral DNA replication or reverse transcription intermediates. These intermediates are easily recognized by the main DNA sensor, cGAS, to induce downstream STING activation. Activated STING recruits TBK1 and IRF3 and further promotes activation and translocation of IRF3 into nucleus to induce the production of type I IFN.

The retinoic acid-inducible gene I (RIG-I)-like receptor (RLR) family members RIG-I and melanoma differentiation-associated protein 5 (MDA5) are crucial for sensing RNA viral infections and for initiating the cascade of signal transduction; this in turn drives the production of type I interferons (IFNs) and the expressions of antiviral genes, thereby inhibiting viral proliferation (Loo and Gale, 2011). During RNA viral infections, RIG-I and MDA5 transmit signals to mitochondrial antiviral signaling protein (MAVS) through a membrane-associated N-terminal caspase recruitment domain (CARD) (Yoneyama et al., 2004, 2005; Kawai et al., 2005). The CARD-like domains of RIG-I or MDA5 combine with the MAVS CARD to activate MAVS, which in turn activates downstream TANK-binding kinase 1 (TBK1)/IκB kinase ɛ (IKKɛ) (Seth et al., 2005). When activated, TBK1 and IKKɛ promote the activation and nuclear translocation of interferon regulatory factor 3 (IRF3), thereby initiating the transcription of target genes that encode type I IFNs (Figure 2; Fitzgerald et al., 2003; Servant et al., 2003; Takahasi et al., 2010).

The host signaling adaptor protein MAVS plays a critical role in driving antiviral innate immunity in response to RNA viral infections (McWhirter et al., 2005). Li et al. (2018) first identified that the OGT-mediated O-GlcNAcylation of MAVS was important for its activation. Subsequently, Song et al. (2019) employed liquid chromatography-mass spectrometry analysis to demonstrate that multiple O-GlcNAcylation sites exist in the amino acid region of MAVS (from 322 to 347). The K63-linked polyubiquitination of MAVS is critical for its activation (Liu et al., 2017a). Compared with that in wild-type MAVS, K63-linked ubiquitination is attenuated in MAVS-7A mutants (in which the serine and threonine residues between 322 and 347 are mutated to alanine); the phosphorylation of IRF3 and the activation of the *Ifnb1* promoter are also inhibited. Thus, multisite O-GlcNAcylation in this region is thought to be essential for the activation of MAVS and its induction of the activation of downstream IRF3 (Song et al., 2019). However, in contrast to the findings presented by Li et al. (2018) and Song et al. (2019), Seo et al. (2020) recently demonstrated that the O-GlcNAcylation of the MAVS 249–257 serine-rich region negatively regulates the host's antiviral immune signaling by interfering with the formation of MAVS aggregates and the interaction between MAVS and tumor necrosis factor (TNF) receptor-associated factor 6 (TRAF6). Compared with the wild type cells, MAVS 249–257 region mutant (7S/T→7A)-transfected cells showed increased levels of IRF3 phosphorylation and IFN-β messenger RNA (mRNA) during Sendai virus (SeV) infection. This suggests that O-GlcNAcylation plays a complex role in the activation of MAVS (Seo et al., 2020). Further systematic studies are needed to determine the differential regulation of immune activation by O-GlcNAcylation.

## Role of O-GlcNAcylation in DNA sensor-mediated signaling pathways

Cellular DNA sensors are mainly absent in melanoma 2 (AIM2), IFN-γ-inducible protein 16 (IFI16), and cyclic guanosine monophosphate–adenosine monophosphate synthase (cGAS) (Paludan and Bowie, 2013). After sensing double-stranded DNA (dsDNA), AIM2 promotes the maturation of downstream pro-inflammatory cytokines (such as IL-1β and IL-18) by assembling the inflammasome complex with apoptosis-associated speck-like protein containing CARD (ASC) and caspase 1 (Burckstummer et al., 2009). IFI16 and cGAS recognize dsDNA, transmit signals to the endoplasmic reticulum-localized stimulator of interferon genes (STING), and promote its oligomerization and activation. When activated, STING recruits and activates TBK1 and IRF3, promoting the translocation of dimerized IRF3 into the nucleus to induce the transcription of type I IFN (Figure 2; Ishikawa and Barber, 2008; Zhong et al., 2008; Unterholzner et al., 2010).

Sterile alpha motif (SAM) domain and histidine-aspartic (HD) domain-containing protein 1 (SAMHD1) is a cellular triphosphohydrolase. It restricts the replication and proliferation of retroviral human immunodeficiency virus type 1 (HIV-1), herpes simplex virus 1 (HSV-1), hepatitis B virus (HBV), and vaccinia virus by regulating the levels of deoxynucleoside triphosphates (dNTPs) in non-cycling cells (Hrecka et al., 2011; Laguette et al., 2011; Hollenbaugh et al., 2013; Kim et al., 2013; Sommer et al., 2016). A recent study has revealed that O-GlcNAcylation plays an important role in regulating the function of SAMHD1. During HBV infection, the OGT-mediated O-GlcNAcylation of SAMHD1 on S93 enhances the stability and tetramerization of SAMHD1 and inhibits the amplification of viral replication. The mechanism for this process may entail O-GlcNAcylated SAMHD1 accelerating the depletion of the intracellular dNTP pool and producing more DNA replication intermediates that are easily recognized by DNA sensors such as cGAS, thereby promoting the activation of antiviral signaling pathways. In particular, it has been found that the S93A mutation does not completely abolish the O-GlcNAcylation level of SAMHD1. This suggests that multiple O-GlcNAcylation sites of SAMHD1 may be involved in the regulation of its antiviral function (Hu et al., 2021).

## Role of O-GlcNAcylation in NLR/TLR/IL-1R/TNFR-induced classical NF-κB pathway

**Figure 3:**
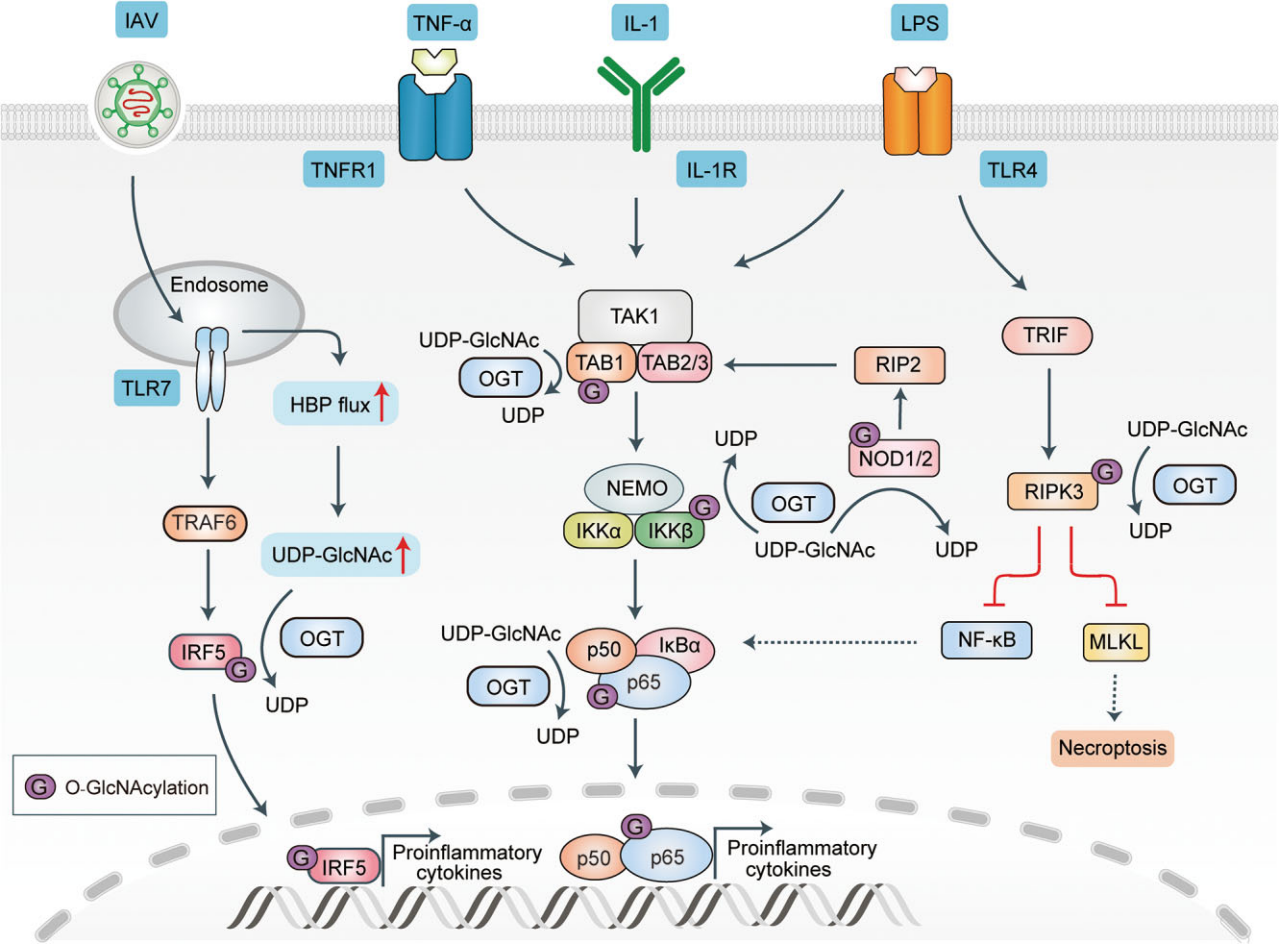
Schematic mode of O-GlcNAcylation in NLR/TLR/IL-1R/TNFR-induced NF-κB pathway. NOD2 induces activation of the TAK1 complex through RIP2 and subsequently activates IKK complex–NF-κB signaling. O-GlcNAcylation of NOD2 stabilizes itself and enhances its signaling capacity. TNFR, IL-1R, and TLR4 all activate TAK1 upon recognition of corresponding ligands and further induce activation and translocation of NF-κB into nucleus. O-GlcNAcylation of TAB1 promotes IL-1-induced auto-phosphorylation of TAK1 and downstream signaling, whereas O-GlcNAcylation of IKKβ is important for TNF-α-induced IKKβ activity. O-GlcNAcylation of p65 promotes expression of LPS-induced inflammatory factors by enhancing transcriptional activity. TLR4 recognizes LPS and transmits signal to RIPK3 through the adaptor protein TIR-domain-containing adapter-inducing interferon-β (TRIF). O-GlcNAcylation of RIPK3 restricts it to an inactive state and inhibits downstream NF-κB- and MLKL-mediated signaling. After TLR7 recognizes IAV, it promotes IRF5 activation via TRAF6–MyD88 signaling. Increased O-GlcNAcylation of IRF5 promotes the production of inflammatory cytokines induced by IRF5.

The nucleotide-binding and oligomerization domains 1 and 2 (NOD1 and NOD2) are members of the canonical NOD-like receptor (NLR) family. This family induces the activation and nuclear translocation of nuclear factor-kappa B (NF-κB) through the TAK1–IKK complex axis (Figure 3; Inohara et al., 2000; Wang et al., 2001; Chen et al., 2009). O-GlcNAcylation exerts a significant effect on the NOD2-induced signal pathway. The OGT-mediated O-GlcNAcylation of NOD2 was first detected using CTD110.6, which is an antibody that detects O-GlcNAc. Treatment with the OGA inhibitor Thiamet G was found to increase the O-GlcNAcylation levels of NOD2, which enhanced both the stability of NOD2 and the activation of NOD2-mediated NF-κB signaling upon muramyl dipeptide (MDP) stimulation (Hou et al., 2016). Similar to NOD2, NOD1 can also be stabilized via O-GlcNAcylation, leading to its high signal transduction ability (Drake et al., 2018). Interestingly, Crohn's disease-associated mutants of NOD2 (R702W and 100fs) have been shown to exhibit low protein stability. Moreover, O-GlcNAcylation can enhance the stability of these mutant NOD2s and promote the downstream activity of NF-κB upon TNF-α and MDP stimulation (Hou et al., 2016; Drake et al., 2018). Thus, O-GlcNAcylation has been demonstrated to promote NOD1/2 signaling by enhancing protein stability, although the O-GlcNAcylation sites remain unclear.

The TAK1-mediated activation of the IKK complex, which consists of IKKβ and IKKα, further induces the phosphorylation and eventual degradation of IκBα, thereby promoting NF-κB signaling (Karin and Ben-Neriah, 2000). TAK1-binding protein 1 (TAB1), TAB2, and TAB3 form a protein complex with TAK1; this is crucial for the IL-1-induced activation of TAK1 (Ninomiya-Tsuji et al., 1999; Ishitani et al., 2003). A recent study reported that the OGT-mediated O-GlcNAcylation of TAB1 promotes the auto-phosphorylation and activity of TAK1, enhancing IL-1-induced NF-κB signaling. Mass spectrometry experiments have been conducted to identify sites of TAB1 O-GlcNAcylation; S395 was identified as the only O-GlcNAcylation site of TAB1. The mutation of S395A blocks the IL-1-induced O-GlcNAcylation of TAB1 and inhibits both kinase activity and the T187 auto-phosphorylation of TAK1, thereby inhibiting downstream NF-κB signaling. This suggests that the O-GlcNAcylation of TAB1 S395 is critical for the full activation of TAK1, as well as the activation of NF-κB (Pathak et al., 2012).

IKKβ, which is the catalytic subunit of the IKK complex, is essential for the activation and nuclear translocation of NF-κB (Karin and Ben-Neriah, 2000). IKKβ has been found to be O-GlcNAcylated in oncogene-induced transformed cells; this requires NF-κB activation to promote glycolysis (Kawauchi et al., 2008, 2009). In the absence of p53, IKKβ–NF-κB signaling and glycolysis form a positive feedback loop, which is crucial for oncogene-induced cell transformation. However, p53 negatively regulates this loop by inhibiting IKKβ. The O-GlcNAcylation of IKKβ at S733 modulates its catalytic activity and promotes a positive feedback loop (Kawauchi et al., 2009). The O-GlcNAcylation of IKKβ S733 has also been shown to promote NF-κB signaling and exacerbate intestinal epithelial inflammation in mice when induced by *Escherichia coli* (AIEC) LF82 infections (Sun et al., 2020). This evidence suggests that the O-GlcNAcylation of IKKβ is critical for oncogenesis and for the development of inflammatory disease regarding the activation of NF-κB signaling.

In mammals, p65 (RelA), RelB, cRel, p50/p105 (NF-κB1), and p52/p100 (NF-κB2) constitute the five members of NF-κB (Ghosh et al., 1998). NF-κB O-GlcNAcylation plays a complex role in regulating inflammatory responses. In vascular endothelial cells, chitosan oligosaccharide (COS) can modulate the lipopolysaccharide (LPS)-induced vascular endothelial inflammatory response by blocking NF-κB nuclear translocation (Liu et al., 2011). The OGT-mediated O-GlcNAcylation of the NF-κB p65 subunit has recently been shown to promote the LPS-induced activation of the NF-κB pathway, whereas the pretreatment of endothelial cells with COS was found to significantly attenuate LPS-induced p65 O-GlcNAcylation levels (Li et al., 2014). This provides new insights into the development of COS-based pharmacological strategies for the treatment of LPS-induced excessive inflammatory responses (Liu et al., 2011; Li et al., 2014). OGT catalytic activity has also been shown to be upregulated via cytosol-specific denitrosylation following the stimulation of LPS, thereby promoting O-GlcNAcylation and the transcriptional activity of NF-κB. Moreover, inhibiting the production of the substrate UDP-GlcNAc with the inhibitor 6-diazo-5-oxonorleucine (DON) has been shown to attenuate the LPS-induced pro-inflammatory production of cytokine (Ryu and Do, 2011). In the placentas of hyperglycemic rats, the O-GlcNAcylation of p65 has been shown to promote the nuclear translocation of NF-κB and pro-inflammatory cytokine production (Dela Justina et al., 2017). Similarly, in mesangial cells, p65 O-GlcNAcylation has been found to directly boost the activation of NF-κB and the expression of inflammation-related factors such as vascular cell adhesion molecule 1 (VCAM-1) and TNF-α. Treatment with GFAT1 inhibitors, O-diazoacetyl-l-serine (azaserine), or DON significantly inhibited p65 O-GlcNAcylation levels and NF-κB signaling (James et al., 2002). Under hyperglycemic conditions, NF-κB p65 can be O-GlcNAcylated at T352, reducing the association between p65 and IκBα and increasing the transcriptional activity of p65. Consistent with this finding, the p65 T352A mutation has been shown to abolish O-GlcNAcylation and inhibit its nuclear translocation and NF-κB signaling (Yang et al., 2008). In addition, Allison et al. (2012) revealed that the O-GlcNAcylation of p65 at T305 is critical for the TNF-α- and etoposide-induced activation of NF-κB. In the heart and endoluminal arteries, the activation of NF-κB is known to be downregulated by increased levels of O-GlcNAc following treatment with GlcN (which is a nontoxic and widely available nutritional supplement) or PUGNAc (which is an inhibitor of OGA) (Xing et al., 2008; Zou et al., 2009). Similarly, another study showed that GlcN and PUGNAc treatments could increase NF-κB p65 O-GlcNAcylation and impair the phosphorylation of p65 at S536 in arterial smooth muscle cells, attenuating the function of NF-κB in signal transduction (Xing et al., 2011). However, the activity of NF-κB signaling also appears to be indirectly regulated by OGT-mediated O-GlcNAcylation. For example, previous studies have reported that glucocorticoid receptor (GR) can interact with and inhibit the functions of NF-κB family members, thereby repressing the transcription of numerous pro-inflammatory genes downstream of NF-κB, such as interleukin-8 (*IL-8*) and intracellular adhesion molecule-1 (*ICAM-1*) (De Bosscher et al., 2003). Nissen and Yamamoto (2000) demonstrated that GR represses the activation of NF-κB by interfering with the S2 phosphorylation of the RNA polymerase II carboxy-terminal domain (pol II CTD). A recent study showed that OGT is a component of a GR-containing multi-protein complex that promotes the GR-mediated transrepression of NF-κB by enhancing the O-GlcNAcylation of pol II CTD, which subsequently impaired pol II CTD phosphorylation induced by TNF-α and further impeded the transcription initiation of *IL-8* and *ICAM-1* (Li et al., 2012). However, the mechanism of crosstalk between the O-GlcNAcylation and phosphorylation of pol II CTD remains unclear.

Receptor-interacting serine/threonine-protein kinase 3 (RIPK3), a member of the RIPK family, is a key signaling molecule in NF-κB signaling and is involved in mixed-lineage kinase domain-like (MLKL)-mediated necrotic cell death (also called necroptosis) (Figure 3; He et al., 2009; Sun et al., 2012; Dondelinger et al., 2014; Silke et al., 2015). Li et al. (2019a) reported that the O-GlcNAcylation of RIPK3 at the T467 site inhibited its activation, while also inhibiting NF-κB and MLKL signaling, avoiding an excessive inflammatory response and necroptosis. In OGT-deficient bone marrow-derived macrophages (BMMs), LPS stimulation has been shown to induce excessive pro-inflammatory cytokine production by inducing NF-κB and extracellular signal-regulated kinase (ERK) signaling. This can be mitigated by treatment with the RIPK3 inhibitor GSK-872. The intracellular expression of OGT has also been shown to inhibit RIPK1/RIPK3-driven NF-κB activity, without affecting IKK complex-driven NF-κB activity (Li et al., 2019a). This suggests that O-GlcNAcylation plays an inhibitory role in RIPK3-mediated inflammatory response and necroptosis.

Regarding influenza A virus (IAV) infections, IRF5 is activated through TLR7–MyD88–TRAF6 signaling and translocates into the nucleus to induce the expression of pro-inflammatory cytokines (Figure 3; Schoenemeyer et al., 2005; Takaoka et al., 2005; Chen et al., 2017). Recent studies have shown that IAV promotes the OGT-mediated O-GlcNAcylation of IRF5 at S430, which is required for the K63-linked ubiquitination of IRF5 and the production of pro-inflammatory cytokines. The IRF5 S430A mutation has been shown to impair IAV-induced pro-inflammatory cytokine production, which suggests that O-GlcNAcylation plays a critical role in IRF5 activation. The overexpression of OGT and IRF5 in peripheral blood mononuclear cells (PBMCs) promoted both inflammatory cytokine expression and viral titers, while knockdown of IRF5 has been shown to have the opposite effect. The authors put forward the possible hypothesis that viral replication is promoted by IRF5 or that excessive production of inflammatory cytokines results in delayed viral clearance, but the exact mechanism has not been elucidated. Notably, the PBMCs from IAV-infected patients have been found to have increased abundances of HBP intermediate metabolites and incorporation of glucose-derived carbons into HBP metabolites, suggesting that IAV may promote the OGT-mediated O-GlcNAcylation of IRF5 by increasing glucose metabolism to promote viral self-replication (Wang et al., 2020).

## Role of O-GlcNAcylation in the JAK–STAT signaling pathway

**Figure 4:**
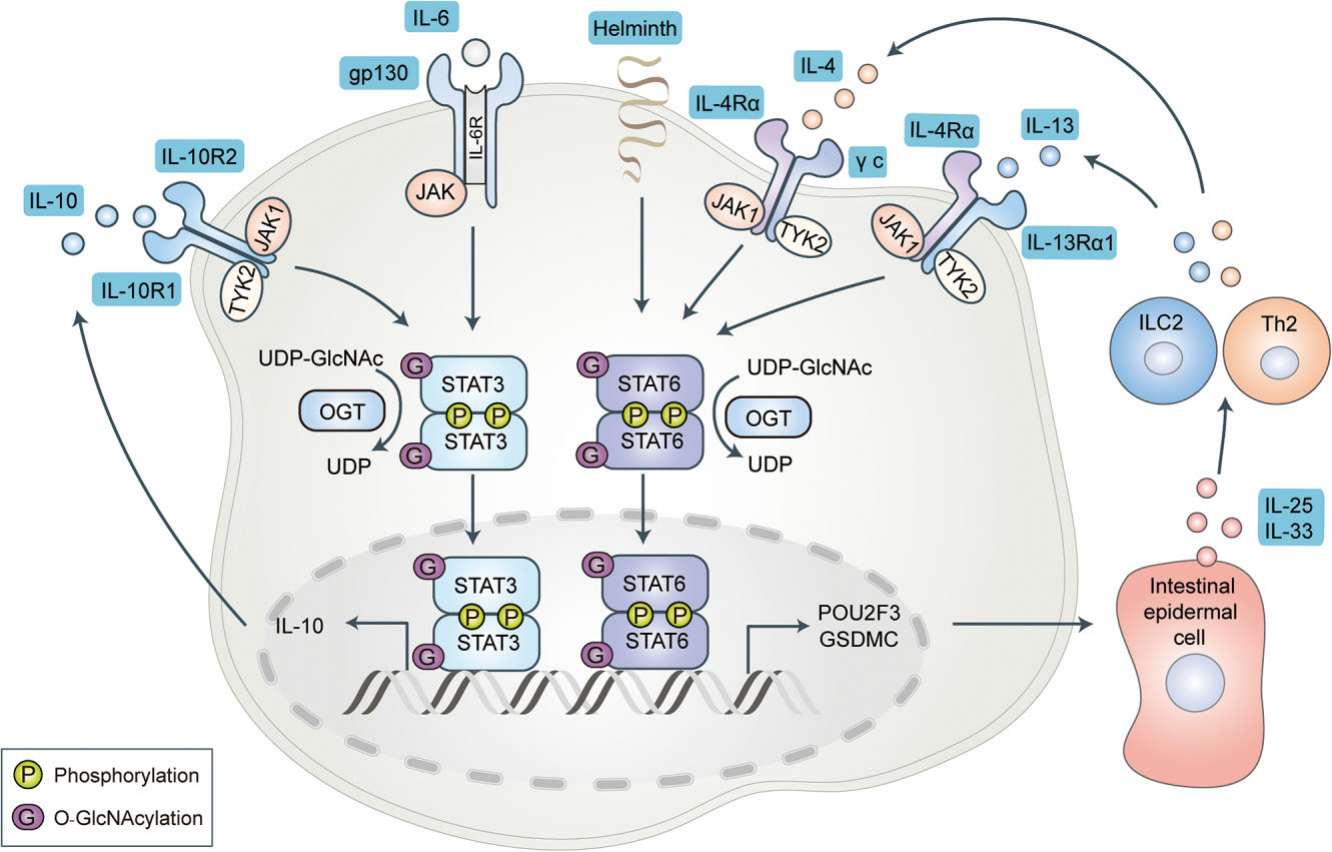
Protein O-GlcNAcylation in the JAK–STAT signaling pathway. IL-6 is recognized by the IL-6 receptor and co-receptor gp130 to activate JAK, which further recruits and activates STAT3. Activated STAT3 forms a dimer and translocates into the nucleus to regulate the expression of target genes, such as IL-10. IL-10 further promotes persistent activation of STAT3 after being recognized by the receptor through autocrine or paracrine pathways. Importantly, O-GlcNAcylation of STAT3 inhibits the activity and expression of target genes. During helminth infection, OGT-mediated O-GlcNAcylation of STAT6 promotes the transcriptional activity of STAT6, which further promotes the transcription of *Pou2f3* and *Gsdmc*. Increased POU2F3 and GSDMC proteins promote IECs to secrete IL-25 and IL-33, which induce ILC2s and Th2 cells to produce type 2 cytokines such as IL-13 and IL-4. IL-13 and IL-4 in turn activate STAT6 to form a loop regulated by OGT-mediated O-GlcNAcylation to regulate helminth expulsion and tolerance.

IL-6 binds to the IL-6 receptor and the co-receptor gp130 to activate Janus kinase (JAK) and signal transducer and activator of transcription 3 (STAT3), followed by nuclear translocation of STAT3 to induce the transcription of target genes such as *IL-10* (Figure 4; Akira et al., 1994; Zhong et al., 1994; Benkhart et al., 2000). Li et al. (2017b) recently revealed that STAT3 is O-GlcNAcylated at T717, which inhibits the transcriptional activity of STAT3 and the expression of downstream genes. The T717 site mutation was found to abolish the O-GlcNAcylation of STAT3 and significantly potentiate the phosphorylation and transcriptional activity of STAT3. STAT3 O-GlcNAcylation is negatively regulated by cullin-3 (CUL3). CUL3 deficiency was found to enhance OGT expression and total protein O-GlcNAcylation levels in macrophages by promoting the stability of nuclear factor-erythroid 2-related factor 2 (Nrf2), which is an OGT transcriptional regulator. Under LPS stimulation, STAT3 phosphorylation levels and the expression of the target gene (*IL-10*) were found to be reduced in CUL3-deficient macrophages compared with wild-type cells. This finding further reveals how STAT3 O-GlcNAcylation regulates the excessive immune-inflammatory activation induced by IL-6 (Li et al., 2017b). The transcriptional activation of STAT6, which is another member of the STAT family, is primarily induced by the binding of IL-4 and IL-13 to their corresponding receptors (Jiang et al., 2000). Zhao et al. (2022) identified that the OGT-mediated O-GlcNAcylation of STAT6 promotes the transcriptional activity of STAT6, and further promotes the transcription of *Pou2f3* and *Gsdmc* during helminth infection (Figure 4). In their work, the authors showed that the STAT6 O-GlcNAcylation-mediated increase in the protein POU2F3 drove the differentiation of tuft cells and promoted the release of the anti-helminth cytokine IL-25. Moreover, they found that GSDMC-mediated unconventional secretion of IL-33 from goblet and Paneth cells was dependent on the formation of GSDMC_N_ pores. Importantly, the secretion of IL-25 and IL-33 by intestinal epidermal cells (IECs) has been shown to promote group 2 innate lymphoid cells (ILC2s) and CD4^+^ T helper 2 (Th2) cells to generate type 2 cytokines (such as IL-13 and IL-4). This initiates the type 2 immune responses for helminth expulsion and tolerance. Subsequently, mass spectrometry has identified eight O-GlcNAcylation sites of STAT6 and confirmed that five sites (S746, T757, S778, S810, and S825) within the transactivation domain (TAD) constitute key O-GlcNAcylation sites. The mutation of all five sites to alanine was found to completely abolish the O-GlcNAcylation levels of STAT6, reduce its transcriptional activity, and decrease the transcription of the STAT6 downstream target genes *Pou2f3* and *Gsdmc*. This further inhibits the production and secretion of IL-25 and IL-33 by IECs, thereby ultimately impairing anti-helminthic type 2 immune responses (Zhao et al., 2022). This finding suggests that the OGT-regulated multisite O-GlcNAcylation of STAT6 is essential for driving type 2 immune responses against helminth infections.

## Crosstalk between O-GlcNAcylation and phosphorylation or acetylation in innate immunity and inflammatory signaling

Analysis of the phosphorylation dynamics of >700 phosphopeptides has revealed that more than half of phosphorylation sites are upregulated or downregulated following an increase in O-GlcNAcylation levels (Wang et al., 2008). This indicates that intimate crosstalk occurs between phosphorylation and O-GlcNAcylation. As serine and threonine residues are required for O-GlcNAcylation and phosphorylation, the extent of competition or cooperation between them depends on the cellular context (Hart et al., 2011). Crosstalk between O-GlcNAcylation and phosphorylation has been reported to regulate signal transduction, transcription, and chronic disease (Butkinaree et al., 2010; Zeidan and Hart, 2010). For example, the phosphorylation of p53 at T155 leads to the subsequent ubiquitination and degradation of p53, while O-GlcNAcylation at S149 hampers T155 phosphorylation and stabilizes p53 (Yang et al., 2006). Upon LPS stimulation, OGT-mediated O-GlcNAcylation of ribosomal protein S6 kinase beta-1 (S6K1) at S489 attenuated its phosphorylation at S418 and T229, thereby inhibiting S6K1 activation. This further suppressed macrophage pro-inflammatory polarization by inhibiting the mTORC1/S6K1 pathway (Yang et al., 2020). In addition, the proto-oncogene c-Myc regulates gene transcription in cell proliferation, cell differentiation, and programed cell death; moreover, T58 can be modified by phosphorylation and O-GlcNAcylation (Chou et al., 1995). In the TNF-α-induced inflammatory response, pretreatment with GlcN or PUGNAc has been shown to increase the O-GlcNAcylation of p65 while decreasing its phosphorylation at S536. This suggests that O-GlcNAcylation may occur near S536 but not necessarily at S536 (Xing et al., 2011). Moreover, the O-GlcNAcylation of STAT3 at T717 inhibits its phosphorylation at Y705 and its downstream signal transduction, which is an evolutionarily conserved mechanism that regulates the biological activity of STAT3 (Li et al., 2017b). In addition, crosstalk between O-GlcNAcylation and acetylation also occurs in innate immunity and inflammatory signaling. There is evidence that O-GlcNAcylation of p65 at T305 may be required for the p300-driven acetylation of p65 K310, which is essential for NF-κB activation (Hoberg et al., 2006). The p65 T305A mutation has been shown to inhibit K310 acetylation without affecting the interaction between p300 and p65. Moreover, the overexpression of the p65 T305A mutant in p65^−/−^ MEFs resulted in decreased NF-κB activity and a high sensitivity to TNF-α-induced apoptosis (Allison et al., 2012). These findings provide a more comprehensive view of the crosstalk that occurs between O-GlcNAcylation, phosphorylation, and acetylation in innate immunity and inflammatory signaling.

## Therapeutics treatment of cancer by targeting O-GlcNAcylation

The aberrant regulation of O-GlcNAcylation has been observed and characterized in many diseases, including diabetes, neurodegeneration, cardiovascular disease, and cancers associated with NF-κB signaling (Copeland et al., 2008; Bond and Hanover, 2013; Wright et al., 2017; Liu and Ramakrishnan, 2021). The NF-κB downstream target gene *IL-8*, which is also known as C-X-C chemokine receptor 8 (*CXCR8*), is a key chemokine that participates in inflammatory responses and cancer development (Ha et al., 2017). IL-8 has recently been shown to increase glucose uptake and consumption regarding the HBP, thereby promoting total protein O-GlcNAcylation in colon and lung cancer cells by regulating the expressions of both GLUT3 and GFAT. Enhanced levels of O-GlcNAcylation are critical for the generation and maintenance of the properties of cancer stem cells (CSCs), tumor metastasis, and relapse after therapy. Moreover, the OGT inhibitor OSMI-1 significantly decreased CSC counts by inhibiting the level of total protein O-GlcNAcylation (Beck and Blanpain, 2013; Shimizu and Tanaka, 2019). Notably, the O-GlcNAcylation of p65 at S322 and S352 promotes NF-κB activity and the downstream expression of the key chemokine CXCR4, which further promotes lung metastasis of cervical cancer cells. Treatment of cells with Thiamet G leads has been shown to lead to intracellular hyper-O-GlcNAcylation and increased expressions of p65 and CXCR4, which can be reversed by treatment with the GFAT inhibitor DON (Ali et al., 2017). Qian et al. (2018) reported that OGT, OGA, and protein O-GlcNAcylation levels were all elevated in pancreatic cancer cells. Moreover, they found that OGT was highly correlated with OGA expression levels. Mechanistically, OGT and OGA are mutually regulated, and OGA can cooperate with p300 to promote the transcription of *Ogt* by activating the transcription factor CCAAT/enhancer-binding protein β (C/EBPβ). Treatment with Thiamet G significantly reduced *Ogt* mRNA levels in mouse pancreatic ductal adenocarcinoma (PDAC) cells, suggesting that high O-GlcNAcylation levels in pancreatic cancer cells depend on the mutual regulation of OGT and OGA (Qian et al., 2018). In another study, O-GlcNAcylation of p65 at S322 and S352 has also been shown to contribute to the induction of anchorage-independent growth in PDAC cells by promoting NF-κB activity. The OGT inhibitor Ac-5S-GlcNAc has also been shown to attenuate intracellular hyper-O-GlcNAcylation and inhibit the proliferation of PDAC cells, suggesting that the pharmacological targeting of OGT may represent a novel treatment for PDAC (Ma et al., 2013). A further study showed that TNF-related apoptosis-inducing ligand (TRAIL) induces apoptosis through the caspase-8 pathway and promotes NF-κB activity by increasing the O-GlcNAcylation level of IKKβ. Treatment with OSMI-1 has been found to block the O-GlcNAcylation of IKKβ, inhibit the activity of NF-κB, and promote the apoptosis of colon cancer cells, providing potential treatment routes for cancer (Lee et al., 2021b).

Cancer cells can reprogram their energy metabolism regarding proliferation, progression, and metastasis by activating the HBP (Fardini et al., 2013). Under high glucose conditions, for example, OGA-associated acetyltransferase activity has been shown to promote the acetylation of pyruvate kinase muscle isoform 2 (PKM2) and further the recruitment of OGT. Moreover, the OGT-mediated O-GlcNAcylation of S362 and T365 in PKM2 inhibits its catalytic activity by blocking its tetramerization, which promotes anaerobic glycolysis and tumor growth. This suggests that PKM2-driven metabolic reprogramming, which is regulated by OGA and OGT, is critical for tumor progression (Singh et al., 2020). Aberrant O-GlcNAc levels can also serve as an indicator for cancer detection (Lee et al., 2021a). Current methods for regulating intracellular O-GlcNAc levels are limited, mainly through the pharmacological inhibition of OGT. Prior studies have reviewed the inhibitors of OGT, including its substrate analogs (UDP, C-UDP, alloxan, BADGP, Ac4-5S-GlcNAc, UDP-S-GlcNAc, and UDP-C-GlcNAc), high-throughput screening (HTS)-derived inhibitors (ST045849, BZX, and OSMI-1), and bisubstrate inhibitors (goblin 1 and goblin 2) (Trapannone et al., 2016; Ju Kim, 2020). However, most OGT inhibitors have been determined to inhibit cancer cell growth based on *in vitro* data; there is a lack of *in vivo* evidence (Slawson and Hart, 2011; Trapannone et al., 2016). Moreover, most OGT inhibitors suffer from limitations such as off-target effects, low efficiency, and an inability to penetrate the plasma membrane (Trapannone et al., 2016). Notably, some recent studies have shown that microRNAs (miRNAs) can inhibit the proliferation and migration of tumors by targeting the 3′-UTR of OGT mRNA to downregulate OGT expression (Liu et al., 2017b, c; Yu et al., 2018). These findings provide new insights into the mechanisms underlying cancer metastasis and may lead to the development of a new strategy for cancer treatment. In summary, more effort should be devoted to the development of compounds that target OGT, potentially in combination with other therapies, to treat cancer.

## Conclusions and future prospects

O-GlcNAcylation participates in the regulation of various cellular and biological functions. Although OGT-mediated O-GlcNAcylation has been observed in many proteins, the mechanisms by which OGT recognizes these diverse proteins remain unclear, as do the mechanisms that regulate OGT activity regarding these proteins. Over the past decade, many studies have aimed to reveal the regulatory roles of O-GlcNAcylation in innate immunity and inflammatory signaling. There is increasing evidence that dysregulated O-GlcNAcylation levels are closely associated with the occurrence and development of diseases and cancer. Balancing and coordinating the complex relationships between energy metabolism, innate immunity, inflammation, and cancer has attracted increasing attention as a research topic. Importantly, it is crucial to identify O-GlcNAcylation proteins in a certain context and to identify the exact sites on these proteins. Detailed analyses could delineate the intricate connections with other PTMs near O-GlcNAcylation sites. In addition, the OGT-mediated O-GlcNAcylation of key signal transduction proteins (such as MAVS and IRF5) is regulated by OGT. These processes are closely related to the activation of antiviral signaling during viral infections. Treatments using pharmacological inhibitors of OGT and OGA could promote the antiviral immune response of host cells to resist virus proliferation and replication by regulating the level of intracellular O-GlcNAcylation. Meanwhile, the occurrence and development of many inflammatory diseases and cancers are often accompanied by abnormally elevated O-GlcNAcylation levels. Moreover, cancer cells can even enhance the metabolic flux of the HBP through metabolic reprogramming to increase the availability of OGT substrates. Therefore, targeting the activity of OGT or OGA using pharmacological inhibitors may constitute a potential therapy for viral infections, inflammatory diseases, and cancers. Overall, O-GlcNAcylation is essential for the precise regulation of innate immunity and inflammatory signaling pathways, and further studies are needed to explore the mechanisms involved. Further preclinical studies are also required to develop novel anti-cancer strategies that target O-GlcNAcylation. Although there are existing compounds that can regulate intracellular O-GlcNAcylation to a certain extent, their safety and effectiveness remain unsatisfactory. Nevertheless, the future development of high-efficiency, low-toxicity drugs targeting O-GlcNAcylation represents an as-yet unsolved route for cancer treatment.

## Notes

**Author contributions:** Y.W., X.F., and S.W. conceived and drafted the manuscript. B.W. and Y.W. drew the figures. F.C. and Z.T. discussed the concepts of the manuscript. S.W., L.Z., and F.Z. provided valuable discussion and revised the manuscript.
